# Isolated left foot drop post aortobifemoral bypass surgery: A case report

**DOI:** 10.1016/j.ijscr.2024.110187

**Published:** 2024-08-17

**Authors:** Amro Hajja, Attas A. Al-Attas, Rasoul Turko, Norah Albugami, Ahmed Almumtin

**Affiliations:** a College of Medicine, Alfaisal University, Riyadh, Saudi Arabia; b King Faisal Specialist Hospital and Research Center, Riyadh, Saudi Arabia

**Keywords:** Aortobifemoral bypass, Foot drop, Common peroneal injury, Vascular surgery, Case report

## Abstract

**Introduction:**

Foot drop is a disorder characterized by weakness in the dorsiflexor muscles of the foot, caused by various pathologies, including neurological, muscular, spinal, and autoimmune conditions. Sometimes, it can be iatrogenic due to direct nerve compression, traction, or ischemia. The occurrence and underlying mechanism of foot drop following aortobifemoral bypass surgery are not well-documented in literature.

**Case presentation:**

A 40-year-old male, with short distance claudication secondary to multi-level lower limb arterial occlusions, mainly, external iliac arteries and superficial femoral arteries. The patient underwent an uneventful aortobifemoral bypass. Post-operatively, the patient developed left sided foot drop with no clear etiology. With intensive physiotherapy, the patient improved and eventually recovered.

**Discussion:**

After excluding other causes of the manifestation, this case could potentially give an insight to a rare postoperative complication following aortobifemoral bypass surgery. Despite a smooth intraoperative course, the patient developed foot drop, a rarely reported complication, suggesting a potential link between the procedure and foot drop.

**Conclusion:**

This case report highlights a rare postoperative complication after aortobifemoral bypass surgery, emphasizing the need for further research to elucidate the direct mechanisms behind this rare occurrence.

## Highlights

• Foot drop can unexpectedly occur after aortobifemoral bypass surgery, even with an uneventful perioperative course.
• Thorough diagnostic evaluations are essential to rule out common causes.
• Careful intraoperative positioning and enhanced postoperative monitoring help prevent and promptly detect this complication.
• After excluding treatable etiology, physical therapy plays a major role in recovery.

## 1 Introduction

Foot drop refers to the condition of diminished strength in the dorsiflexor muscles of the foot, most caused by a lesion of the common peroneal nerve [1]. The nerve is susceptible to compression and injury in the area where it encircles the fibular neck. This is because it lies superficially and makes a transition beneath the lateral compartment muscles, making it more vulnerable to external trauma and swelling [2,3].

The causes of foot drop are diverse and can stem from neurological, muscular, spinal, autoimmune, and neural injuries, which can originate anywhere along the neuromuscular pathway [[1], [2], [3], [4]]. Iatrogenic injury often occurs during procedures like osteosynthetic or arthroscopic surgery [5]. Peroneal nerve injuries following vascular procedures have been documented in multiple cases [[6], [7], [8]]. Of these cases, two were reported after aortobifemoral bypass surgery: the first one was caused by sciatic nerve compression due to a localized hematoma around the groin area [8], and the other resulting from ischemic peroneal nerve injury [6]. In this case, we report another occurrence of isolated left foot drop following aortobifemoral bypass surgery in a young patient. This case report has been reported in line with the SCARE criteria and PROCESS guidelines [9,10].

## 2 Case presentation

**Fig. 1:**
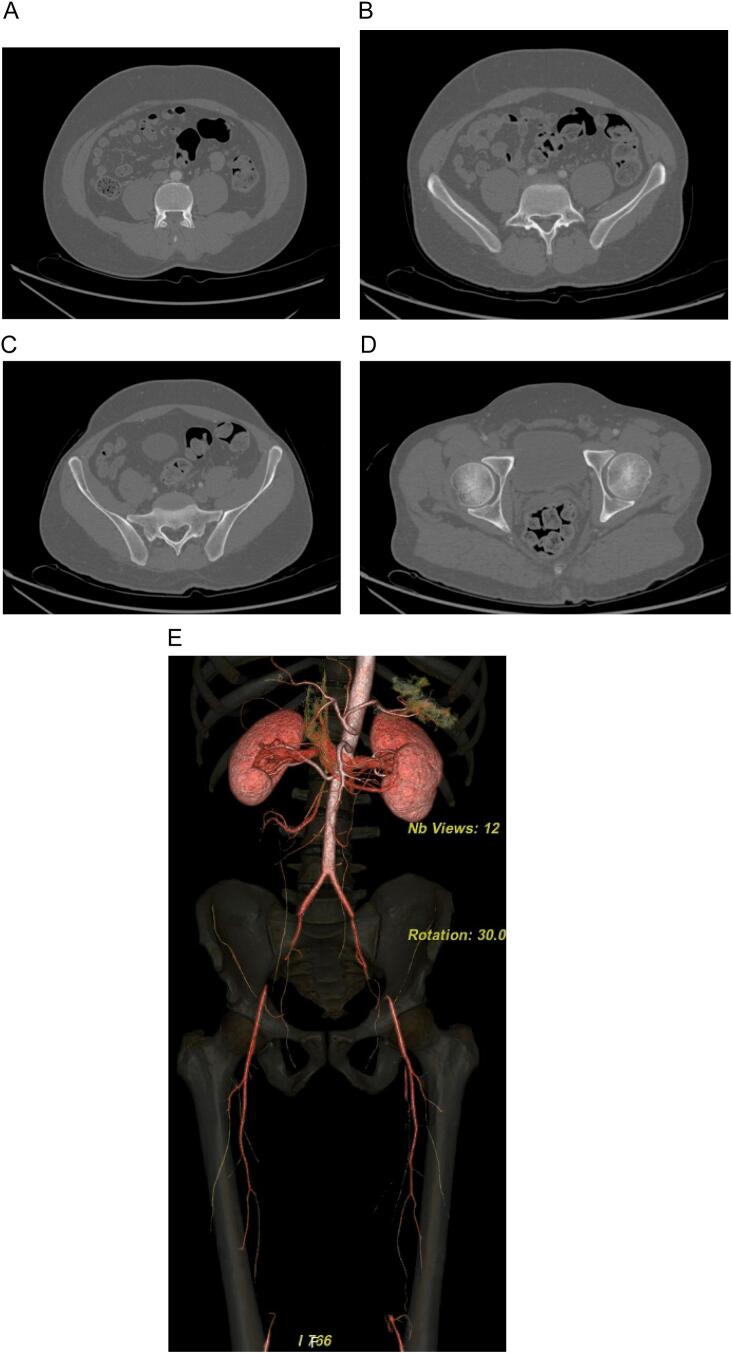
Figure 1: CT angiography of lower abdominal aorta and lower extremities. 1A: Patent distal aorta, just proximal to the bifurcation. 1B: Patent bilateral common iliac arteries. 1C: Occluded bilateral external iliac arteries, and patent bilateral hypogastric arteries. 1D: Reconstruction of flow in both common femoral arteries through collaterals. 1E: 3D reconstruction of his lower limb angiography showing occluded external iliac arteries and proximal two thirds of both superficial femoral arteries.

A heavy smoker 40-year-old male with peripheral vascular disease presented to the clinic with worsening claudication, tolerating no more than 100 m of walking, and a non-healing right 5th toe ulcer. His past medical history includes type 2 diabetes mellitus and a 36-pack-year smoking history. On examination, he had no palpable femoral pulses with monophasic distal Doppler signals bilaterally. He was neurologically grossly intact, ambulating without support, with preserved sensation, and normal muscle strength bilaterally. Based on the radiological investigations, including Doppler US and CT angiography, the patient was found to have bilateral external iliac artery occlusion. The internal iliac arteries and common iliac arteries were patent. The common femoral arteries were also patent with run-off to profunda femoral arteries and to distal superficial femoral arteries through collaterals (Fig. 1A, B, C, D, E). The popliteal arteries and below-knee run-off arteries had diffuse disease. After counseling the patient, we decided to proceed with surgery after obtaining a preoperative workup and necessary surgical consent.

### 2.1 Surgery

After general anesthesia and endotracheal intubation, routine prepping and draping were completed, and we placed the patient in a supine position. A midline laparotomy incision was made, transperitoneal approach was used to expose the abdominal aorta. An aortobifemoral bypass was conducted using a 16–8 mm bifurcated Dacron graft. After infra-renal clamping, proximal anastomosis was completed in an end-to-side fashion using 4-0 polypropylene within 18 min. Afterwards, the clamp was relocated to the graft. Distal anastomosis was completed on both femoral arteries using 6–0 polypropylene sutures. The internal iliac arteries were preserved. The whole operation was conducted over 4 h, during which blood pressure was maintained within an acceptable range before, during, and after aortic clamping. Doppler signals were biphasic on anterior and posterior tibial arteries by the end of the procedure. Two groin drains were inserted and fixed to the skin. The urine output was satisfactory, and the estimated blood loss was 750 ml. He was kept overnight in the surgical ICU, where he received no inotropes or any specific measures.

### 2.2 Post-operative course

Prophylactic heparin injections were resumed eight hours postoperatively, and pain was well controlled. He maintained good hemodynamics. However, twelve hours after the procedure, the patient started to complain of inability to dorsiflex his left foot.

**Fig. 2:**
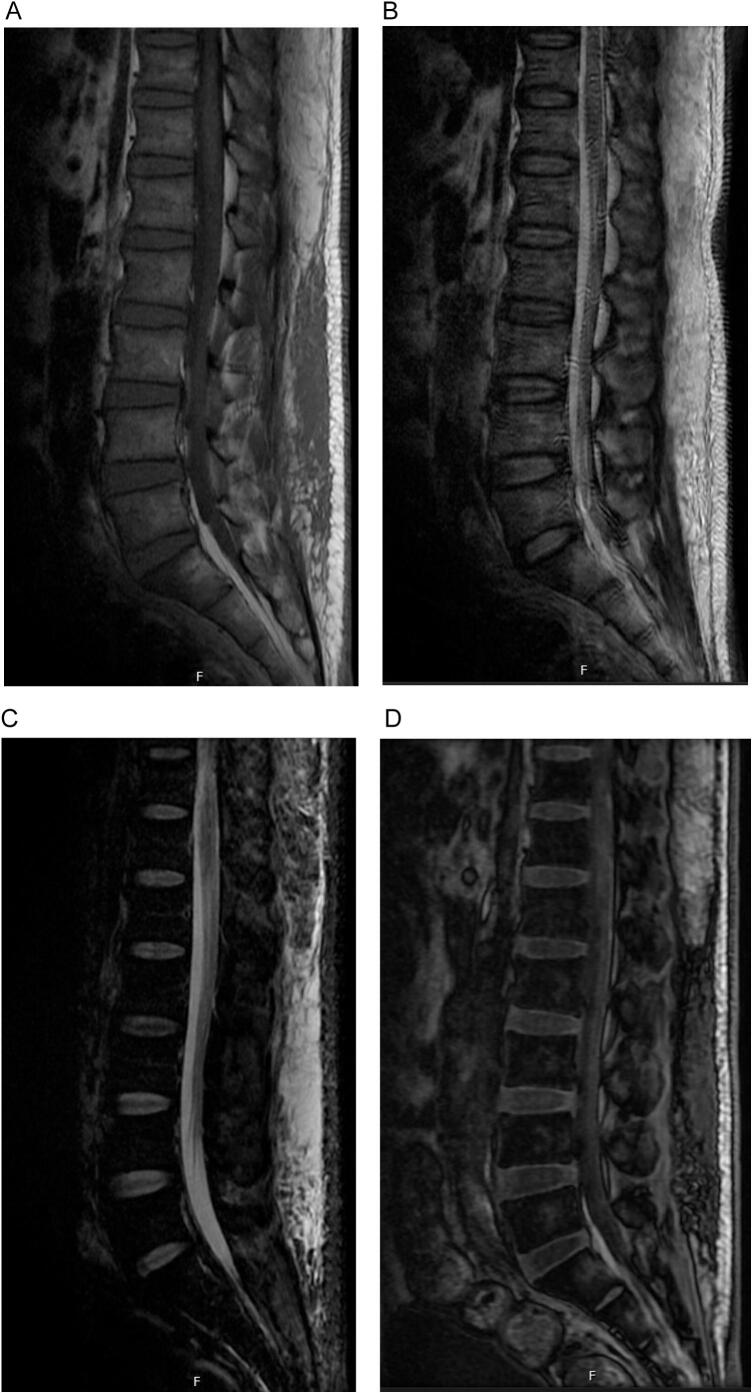
Figure 2: MRI spine. 2A: Sagittal T1 graded with no evidence of disc prolapse. 2B: Sagittal T2 graded with no evidence of local compression on the cord, disc prolapse or subluxation. 2C: Sagittal STIR (short Tau inversion recovery). 2D: Sagittal 3dDualEcho.

On examination, the patient was vitally within normal limits. He had average muscle bulk and tone in the lower limbs bilaterally. There was numbness and reduced light touch on the left foot's dorsum. Left achilleas reflex was zero. The power was 0/5, affecting dorsiflexion and eversion of the left foot. The foot was otherwise warm to touch and with good biphasic distal signals. Over the next two days, the patient exhibited spontaneous symptomatic improvement with a power of 2/5. A spine MRI was done to rule out any spine pathology but was unremarkable (Fig. 2A, B, C, D). A nerve conduction study (NCS) was reported to exhibit significant changes indicative of severe chronic sensory motor axonal peripheral polyneuropathy.

Consequently, the patient was discharged home and referred to physiotherapy. Two months later, the patient returned to the clinic, where noticeable clinical improvement in the foot drop was observed. A repeat NCS showed normal velocity in the left peroneal nerve. However, the abnormal motor and sensory responses from the left lower extremity pointed to a proximal lesion in the lumbosacral region.

## 3 Discussion

Our case presents a unique instance of an isolated left foot drop following aortobifemoral bypass surgery. The patient underwent surgical intervention due to a bilateral external iliac artery occlusion. Despite the smooth intraoperative course, an unexpected complication surfaced within the first 24 h—a new-onset left foot drop. Unilateral left foot drop can be due to a wide range of etiologies, including common peroneal neuropathy, being the most common cause. Common peroneal nerve injury occurs due to compression at either the fibular head or popliteal fossa or in prolonged lithotomy or crossed legs position [11,12]. Our patient was in a supine position with uncrossed legs and had no compression applied to any of the sites mentioned. Moreover, NCS showed normal velocity in the common peroneal nerve, which rules out common peroneal neuropathy.

Another well-recognized etiology is sciatic neuropathy due to compression between the piriformis muscle and pelvic bones or due to gluteal region hematomas secondary to intragluteal injections commonly seen in regional anesthesia. This was excluded, as the patient was under general anesthesia and had no recent history of gluteal injections [13].

Lumbosacral plexopathy (LSP) is another etiology due to several causes, including pelvic surgeries due to mechanical compression of a hematoma or because of pelvic ischemia. In LSP, patients predominantly present with back pain extending to a unilateral thigh with associated muscle weakness, buttock claudication, absent or reduced deep tendon reflexes in the entire leg, sensory loss in dermatomal pattern, and weak anal tone [14]. Our patient is pain-free with an absent unilateral left Achilles tendon reflex, impaired dorsiflexion, and eversion of the left foot. The rest of the left leg muscles have normal power. The anal tone is intact. Sensory loss is restricted to the left foot dorsum. MRI did not reveal any characteristics of LSP. However, NCS was suggestive of a proximal lesion in the lumbosacral plexus. Moreover, ischemic injury to the peroneal nerve has also been identified as a contributing factor to foot drop, as demonstrated in this case [6]. Other less common causes include upper motor neuron lesions at the cerebral cortex and corticospinal tract levels.

The identification of such rare complications emphasizes the necessity for meticulous intraoperative positioning and during the postoperative period. This case suggests that clinicians should consider the possibility of lumbosacral plexus involvement when patients present with new-onset foot drop post vascular surgeries, even in the absence of common risk factors.

Enhanced postoperative monitoring and early diagnostic interventions, such as NCS and MRI, can facilitate prompt identification and management of such complications, potentially improving patient outcomes.

Our case suggests a potential link between aortobifemoral bypass surgery, and the development of foot drop secondary to LSP, which is an atypical presentation of a rare complication of this procedure. It also highlights the need for heightened clinical awareness, thorough differential diagnosis examination, and the utility of diagnostic interventions. Enhanced postoperative monitoring and a proactive approach can significantly improve patient outcomes by facilitating prompt identification and management of such complications.

## 4 Conclusion

Our case expands the understanding of neurological complications post aortobifemoral bypass by introducing a unique presentation of unilateral foot drop. This case not only highlights an atypical manifestation but also emphasizes the diversity of potential mechanisms, including ischemic and compressive etiologies. These findings underscore the necessity for ongoing research and multicenter collaborations to better elucidate the direct mechanisms behind this complication.

## Consent

Written informed consent was obtained from the patient for publication of this case report and accompanying images. A copy of the written consent is available for review by the Editor-in-Chief of this journal on request.

## Ethical approval

King Faisal Specialist Hospital and Research Center waived the need for IRB approval due to the absence of patient identification in the study's enrolled participant.

## Funding

The research paper did not receive any funding.

## Author contribution

Amro Hajja, Literature review and data collection.

Attas A. Alattas, Literature review and revision.

Rasoul Turko, Manuscript dictation.

Norah Albugami, Materials and revision.

Ahmed Almumtin, overall supervision and editing.

## Guarantor

Amro Hajja.

## Conflict of interest statement

The authors have no competing interests.
